# Primary mediastinal seminoma with florid follicular lymphoid hyperplasia: a case report and review of the literature

**DOI:** 10.1186/s13000-021-01137-9

**Published:** 2021-08-21

**Authors:** Charlotte Holmes, Peh Sun Loo, Sion Barnard

**Affiliations:** Freeman Hospital, Newcastle upon Tyne, UK

**Keywords:** Primary mediastinal seminoma, Mediastinal seminoma, Seminoma, Florid follicular hyperplasia, Thymic mass, Mediastinal mass, Thymectomy

## Abstract

**Background:**

First described in 1955 Primary mediastinal seminomas are rare. Only 1–4% of mediastinal tumours are germ cell tumors; majority of which are teratomas. They typically present in men aged between 20 and 40 years. Very few cases are reported in the literature. Florid follicular lymphoid hyperplasia can obscure the malignant cells and is a rarer finding still. We present a rare case of a 48 year old man with a primary mediastinal seminoma with florid follicular lymphoid hyperplasia; found following excision of a clinically presumed thymoma.

**Case presentation:**

A 48 year old man was referred for excision of a thymic mass. The presumed diagnosis was a thymoma; following preoperative investigations. The mass was incidentally found on a radiological imaging. However, the patient did report mid-sternal discomfort on lying flat and breathlessness. The patient underwent a thymectomy via a partial median sternotomy with good recovery. Histological assessment was that the mass was in fact a primary mediastinal seminoma with florid follicular lymphoid hyperplasia. A primary testicular malignancy was excluded and the patient required no further oncological treatment.

**Conclusions:**

Only 11 cases have previously been reported of primary mediastinal seminoma with florid follicular lymphoid hyperplasia. Although rare, a primary mediastinal seminoma should be considered as a differential diagnosis for presentations with a thymic mass. Tumour markers can be helpful, however are only positive in third of cases. Ultrasound imaging of the gonads is essential to exclude a primary gonadal lesion. Pure seminomas are radiotherapy and chemotherapy sensitive however the mainstay treatment of primary mediastinal seminomas remains surgical excision. Radiotherapy is reserved postoperatively for incomplete surgical margins.

## Introduction

Seminomas are a type of germ cell tumour that usually occur in the reproductive organs. Extragonadal germ cell tumours, on the other hand, typically present in the midline structures, such as the retroperitoneum and mediastinum [1–3]. Primary mediastinal seminomas are rare and were first described by Woolner et al. in 1955 [2, 4]. Only 1–4% of mediastinal tumours are germ cell tumours; with seminomas being the second most common to teratomas [1, 2]. Seminomas are slow growing and are often large in size at time of diagnosis with infiltration of local structures [1, 3].

Histologically, primary mediastinal seminomas with florid follicular hyperplasia are even rarer. At times, secondary accompanying features such as florid lymphoid hyperplasia or cystic changes can be quite pronounced, thereby obscuring the malignant cells. Given that these are morphologically indistinguishable from their gonadal counterparts, it is therefore prudent to exclude the possibility of seminoma metastasising to the mediastinum from the gonad [5].

In general, primary mediastinal seminomas have a good prognosis [1, 3, 6, 7],typically present in men of age 20–40 years^3.^, often asymptomatic and found incidentally in 20–30% of cases. Our case is of a 48 year old gentleman with an incidental finding of a primary mediastinal seminoma with florid follicular lymphoid hyperplasia who had undergone an excision of a clinically presumed thymoma.

## Case report

### Clinical history

A 48 year old gentleman was referred for excision of a thymic mass after multidisciplinary team (MDT) discussion. The mediastinal mass was incidentally found via investigation for abdominal pain which then led to Computed tomography (CT) of the chest. Retrospectively, a CT coronary angiogram from 3 years prior also showed the mass; which was not reported at the time. The mass was relatively unchanged in appearance on the latest CT. The patient reported symptoms of mid-sternal discomfort when lying flat associated with breathlessness. There were no symptoms or signs of myasthenia gravis.

He had a past medical history of raised body mass index (BMI) and previous right inguinal hernia repair but was not on any regular medication. He was an ex- smoker, works as a window cleaner and lives with his wife and child. Examination was unremarkable apart from a BMI of 48.7.

**Fig. 1:**
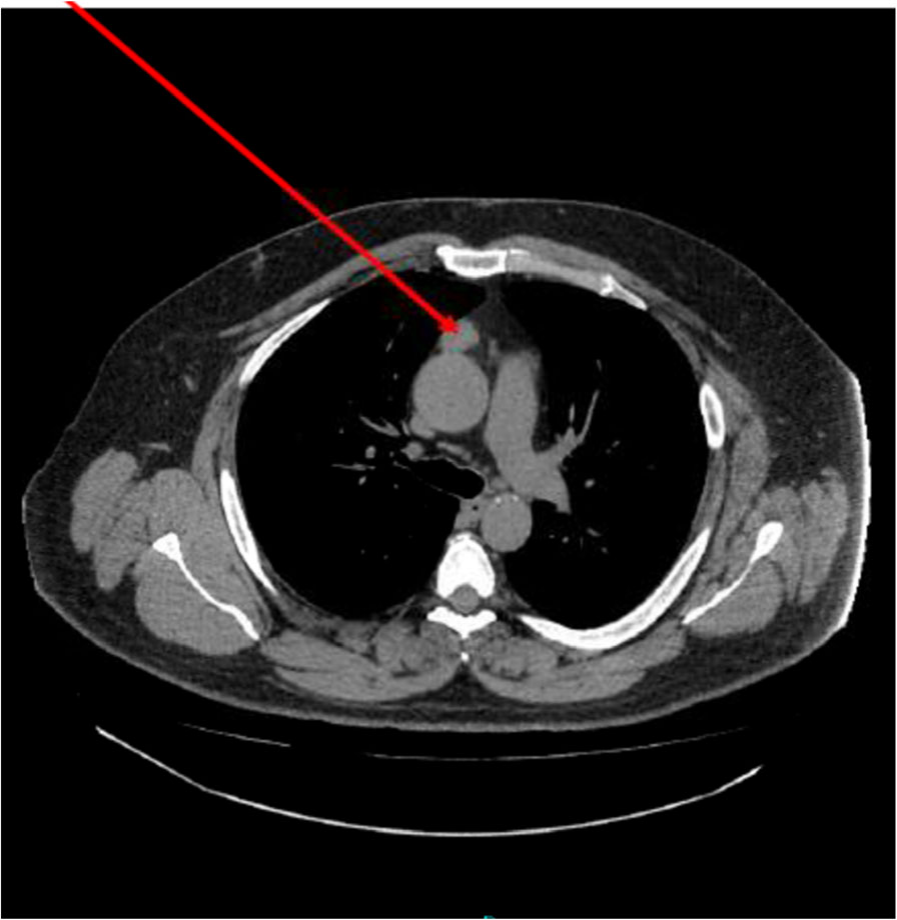
Arrow indicating anterior mediastinal mass on CT imaging

CT imaging, from 5 month prior to his operation, showed a slightly lobulated and triangular lesion 40 mm (craniocaudal) × 23 mm (transverse) × 16 mm (anteroposterior) in size in the anterior mediastinum, just anterior to the aortic arch (Fig. 1). An MRI thorax a month following the CT showed a soft tissue lesion 34 mm (craniocaudal) × 25 mm (transverse) × 18 (anteroposterior) in size.

### Operative details

Due to limited access with thoracoscopy, due to increased BMI, and the risk of conversion a partial median sternotomy was the approach of choice. Intraoperatively, the appearances were of a 6 cm lesion. It was larger than expected, being adherent to the pleura and partially to the pericardium but without evidence of direct invasion. The tumour was excised with a combination of blunt and sharp dissection from its inferior aspect up to the left innominate vein. After exposing the vein and clipping and cutting tributaries, the upper mediastinal dissection continued. The superior horns of the thymus were dissected out of the neck brought down into the mediastinum, and excised. The mass was dissected free from the right pleura, involving adhesionolysis and then as far laterally to the left as the mediastinal fat allowed. Given that there was a large amount of mediastinal fat, a good margin clearance was achieved. Two drains were placed; one into the right pleura which was open and another to the left of the mediastinum. The sternum was closed with stainless steel wires and the wound closed in layers.

### Pathology

**Fig. 2:**
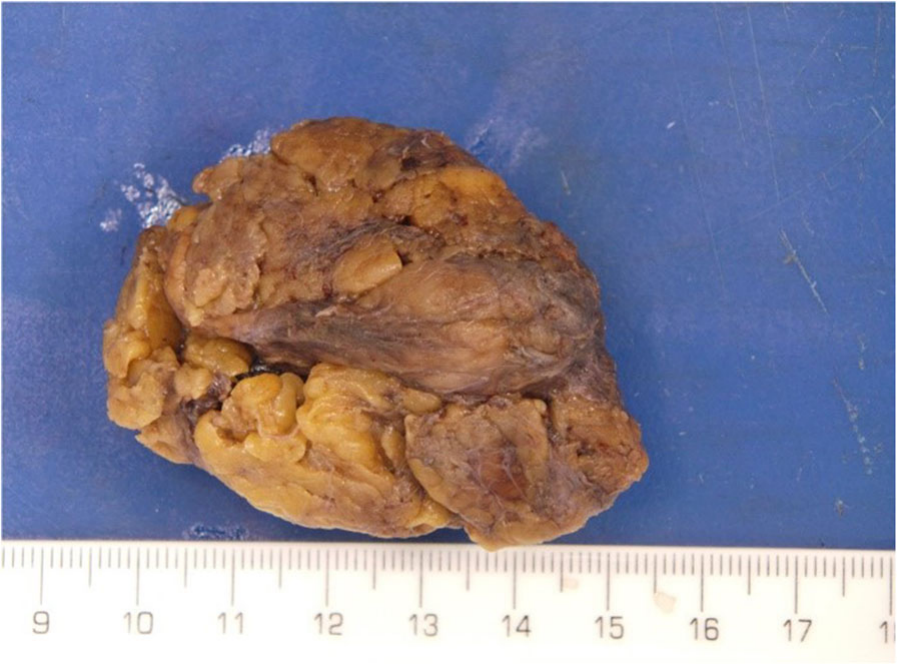
Macroscopic specimen of primary mediastinal seminoma intact and in cross section

**Fig. 3:**
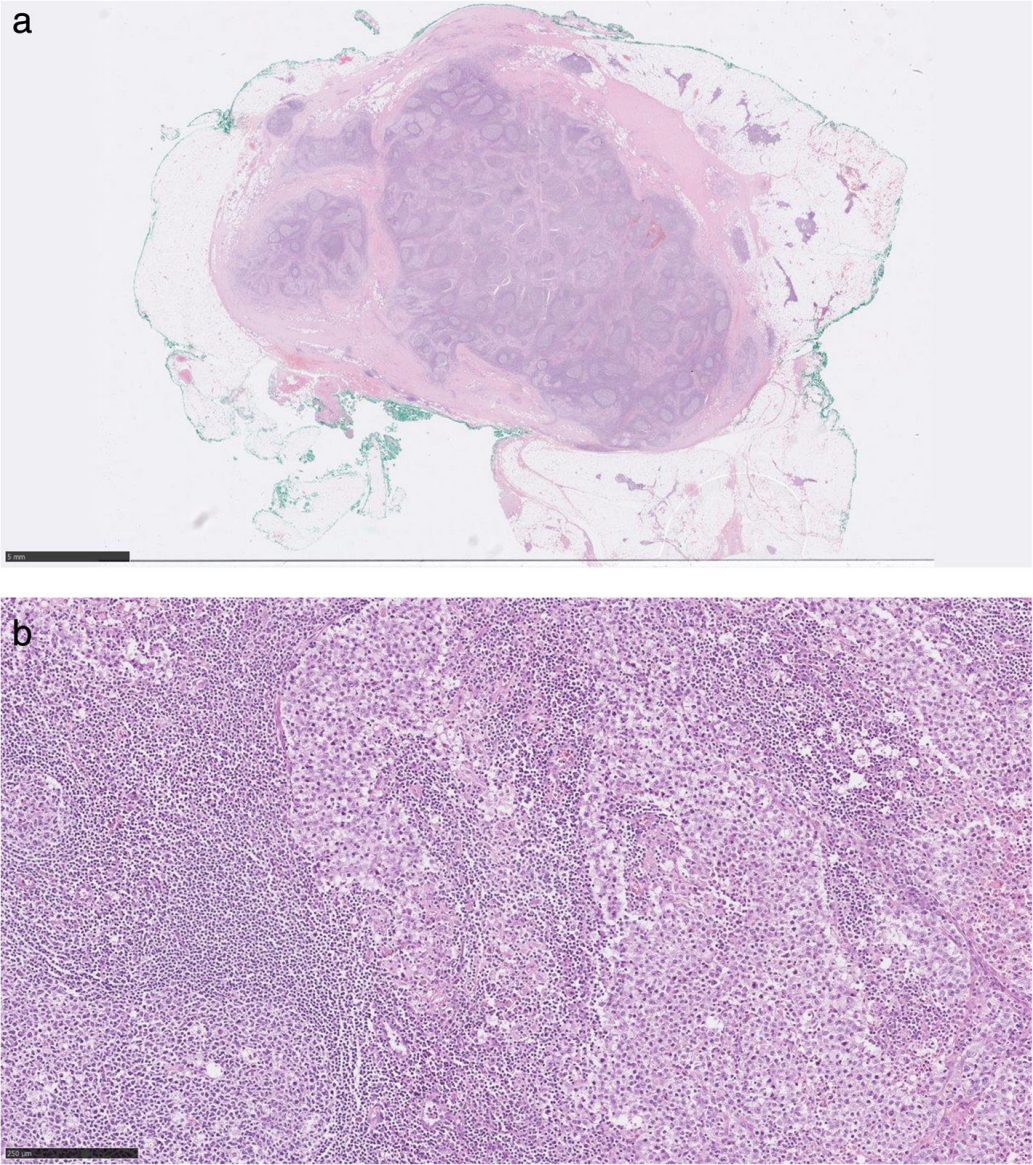
H&E staining × 0.5 magnification (top) and high-resolution × 10 magnification (bottom)

Macroscopically, the lesion was a smooth, lobulated 60 × 45 × 30 mm mass which appeared partly surrounded by fibroadipose tissue (Fig. 2). Serially slicing showed a well circumscribed pale fleshy, cream nodule of 29 × 18 × 25 mm. Histologically, the lesion was a fairly circumscribed, lobulated tumour composed of discohesive round to polygonal cells with indistinct cell borders, clear cytoplasm and prominent nucleoli associated with a background of prominent follicular hyperplasia. These discohesive cells are reminiscent of classical seminoma cells. In addition, in the background within the surrounding adipose tissue around this tumour, there are residual foci of thymic remnant tissue (Fig. 3).

**Fig. 4:**
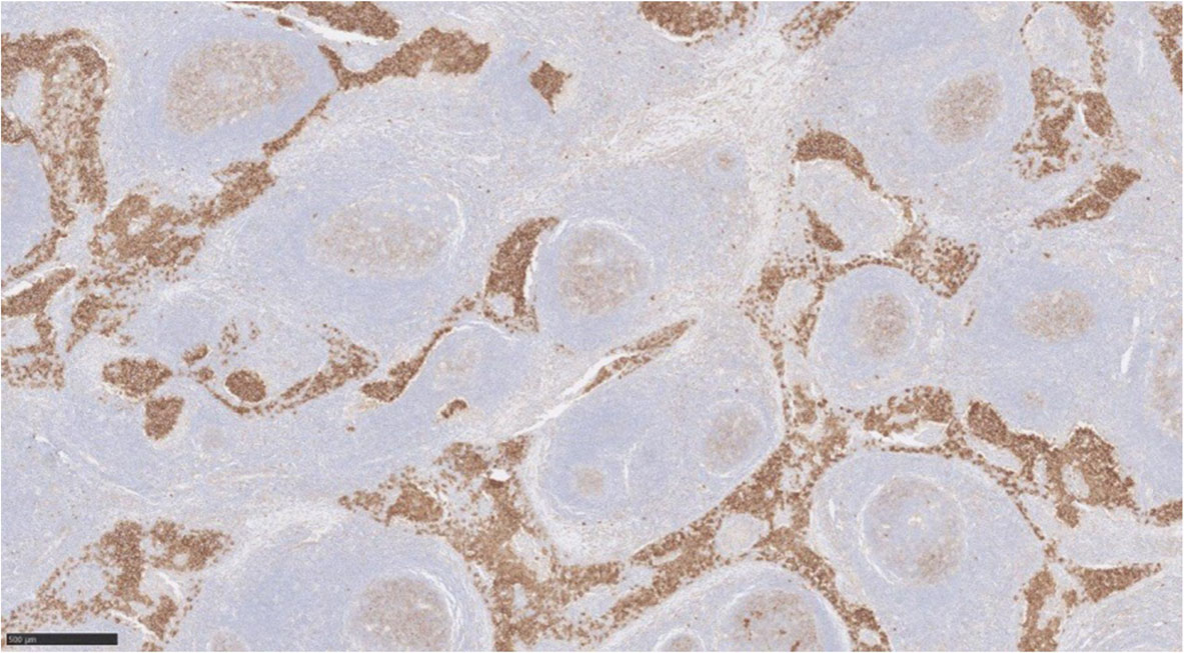
Immunohistochemistry CD117, image × 5 magnification, confirming the diagnosis of seminoma

**Fig. 5:**
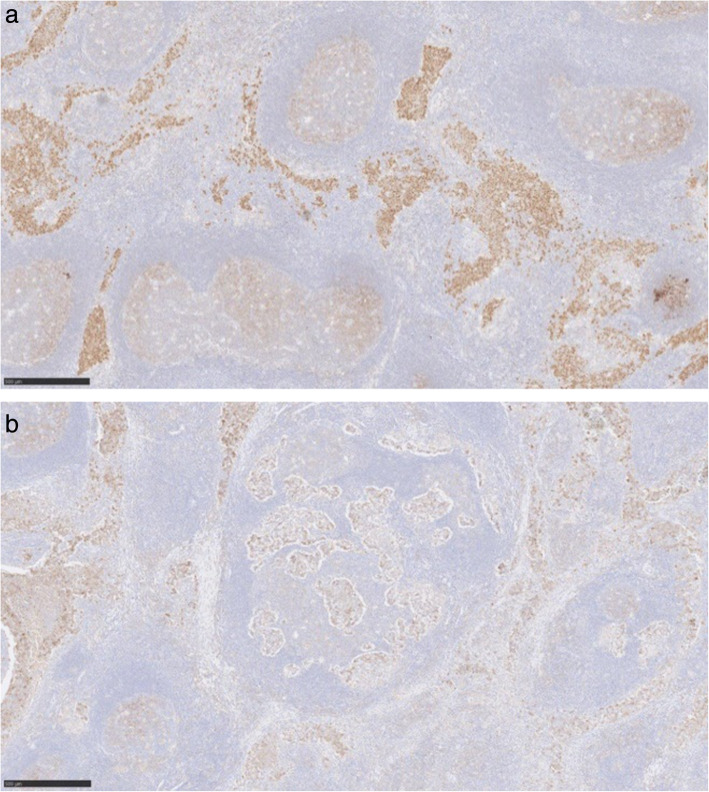
Immunohistochemistry Oct4 (left) and PLAP (right), both images × 5 magnification, confirming the diagnosis of seminoma

**Fig. 6:**
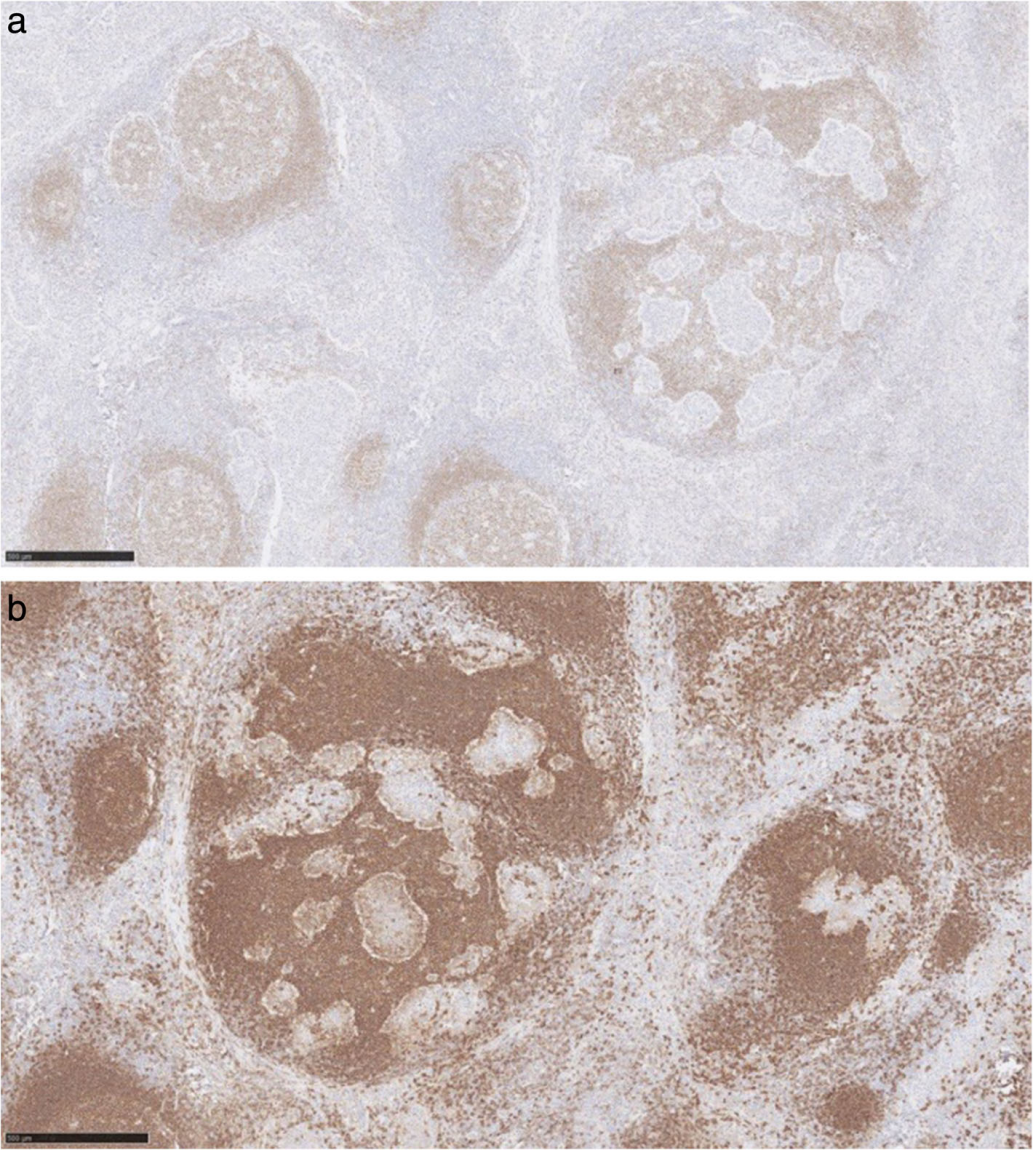
Immunohistochemistry PAX-8 (left) showing the lymphoid follicles with germinal centres and CD20 (right) showing background B lymphocytes with germinal centres. Both images are × 5 magnification

**Fig. 7:**
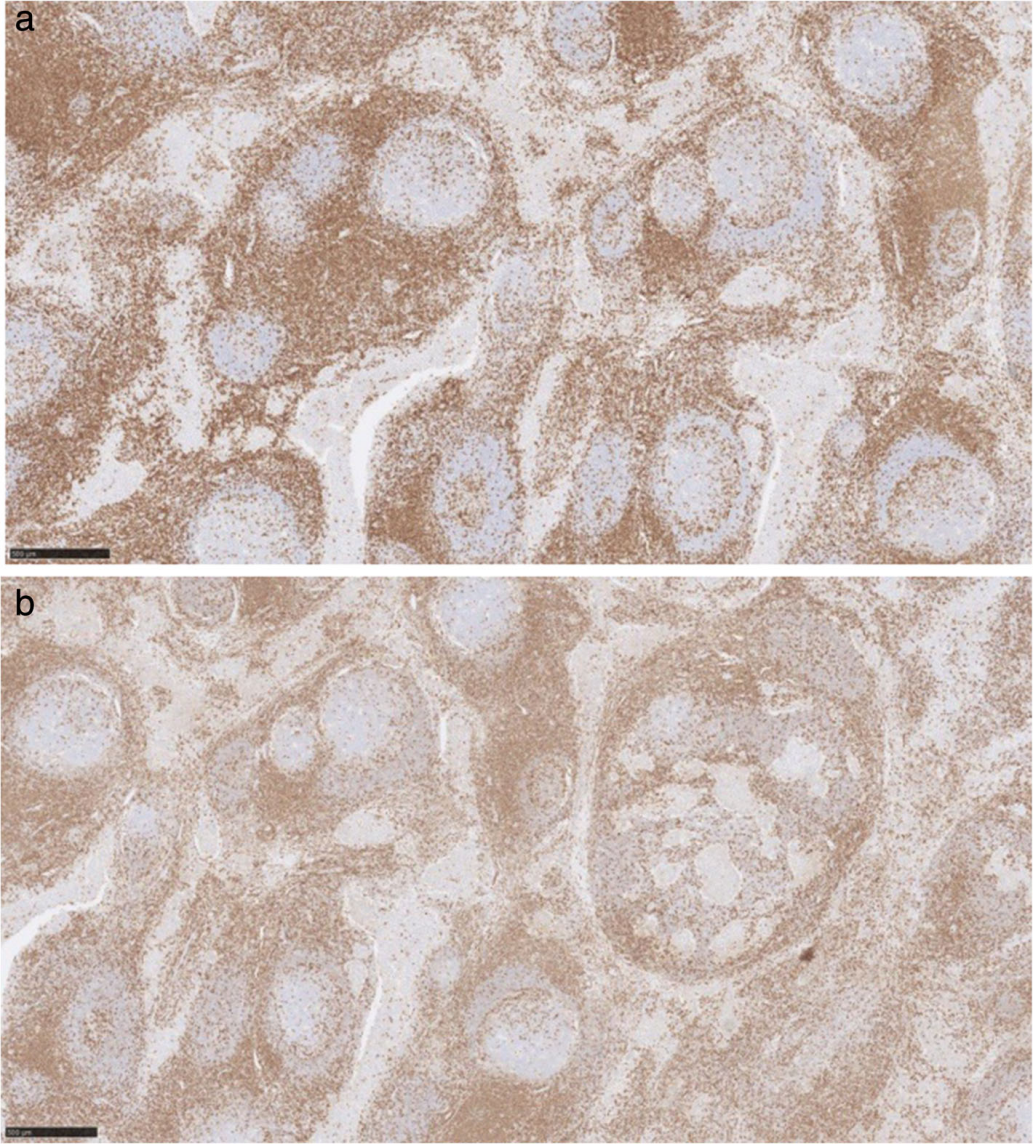
Immunohistochemistry CD3 (left) CD5 (right), both images × 5 magnification, depict the background T lymphocytes

**Fig. 8:**
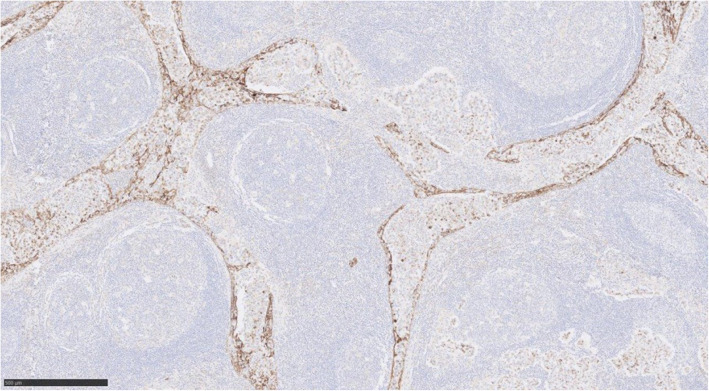
Immunohistochemistry CKAE1/3, image × 5 magnification, shows the perinuclear dot-like pattern in the seminoma cells

Immunohistochemically, the discohesive cells are positive with CD117 (Fig. 4), Oct4 and PLAP (placental alkanie phosphatase) (Fig. 5). CD20 highlights the background B lymphocytes within the germinal centres. Similarly PAX-8 highlights the lymphoid follicles with germinal centres. (Fig. 6) Both CD3 and CD5 highlight the background T lymphocytes in the interfollicular areas as well as in between the tumour cells (Fig. 7) TdT-terminal deoxynucleotidyl transferase is negative in the tumour but is positive in the background residual thymic tissue. CKAE1/3 shows perinuclear dot-like pattern in the seminoma cells (Fig. 8).

Overall, the appearances are consistent with a primary mediastinal seminoma with florid follicular lymphoid hyperplasia. In several foci, the lobules of lymphoid follicles abuts painted margin. Focally, particularly on the immunostain CD117, there are scattered seminoma cells infiltrating right into the paint surface. Therefore, the completeness of excision could not be guaranteed.

### Follow up

The patient had a routine overnight stay in our intensive care unit, and his drains were removed on day one. He was stepped down to the ward and once analgesia was adequate was discharged on postoperative day three. Post discharge, his wound healed well and the ongoing pain secondary to sternotomy eventually settled. However, he reported an ongoing breathlessness without any explainable CT or Chest X-ray findings. His breathlessness is being investigated by his respiratory physician. Repeat Pulmonary function tests and a ventilation perfusion (V/Q) scan were normal. An echocardiogram is currently awaited.

Following histology results, the patient discussed in the Urology MDT and subsequently referred to an oncologist, who suggested tumour markers namely beta human chorionic gonadotropin (βHCG), alpha-fetoprotein (αFP) and lactate dehydrogenase (LDH) levels which were negative and a repeat staging CT which was normal. The patient had a testicular ultrasound at the time of his original presentation, prior to his surgery, which excluded a testicular primary. A repeat positive emission tomography (PET) scan did not show any lesions either.

Given the normal staging CTs, ultrasound, PET scan and negative tumour markers, the MDT decision was for active surveillance.

## Discussion

Mediastinal tumours are most commonly thymomas, neurogenic tumours or benign cysts, altogether accounting for 60% of cases [8].. Primary mediastinal seminomas are considered rare entities, being most common in men in the third to fourth decade of life. However, there have been cases arising in women [3, 9]. The role of tissue diagnosis with fine needle aspiration or core biopsy prior to surgery is controversial. It is thought that biopsying these lesions results in a high risk of tumour seeding, and ultimately recurrence. It is also common to proceed to surgery after biopsy; therefore negating the need for this investigation in most cases. Particularly, in cases such as ours where the tumour is large and or the patient has symptoms where surgical excision is required [10]. As in our case, pre-operative assessment with CT imaging to assess anatomy and vascular involvement and PET scanning to investigate for malignancy prior to surgery is imperative. MRI is also a useful modality to look for local invasion [7, 10].

Tumour markers such as βHCG and αFP are tumour markers which can be used diagnostically as well as follow up for recurrence. αFP is not secreted by primary mediastinal seminomas and therefore would either indicate a mixed germ cell tumour or testicular primary [2, 6]. βHCG is only secreted in approximately a third of primary mediastinal seminoma cases [6]. Napieralska et al. 2018 in a cohort study found a raised βHCG in 38% of their cases [2]. As in our case, testicular primaries should be excluded with examination and ultrasound, however, they are very rarely present [6, 11].

A review of the literature for primary mediastinal seminoma shows few recent case reports. The case series by Napieralska et al. looked at a single centers cases from 1983 to 2014. In this 31 year period they had 16 cases; 6 found incidentally after surgery as in our case [2]. Petrova et al. in 2019 reported a single case however the patient has significant pericardial and pleural effusion, and the lesion was biopsied for diagnosis and underwent chemotherapy treatment with good response [3]. A very similar presentation to a case reported in 2017 by Thompson et al. where an obstructing mediastinal seminoma was treated with chemotherapy [12]. A case study published in 2010 by Chen et al. described a similar case to ours in that the mediastinal seminoma was diagnosed following surgery for a presumed thymomas however the patient was female, which is very rare and at the time the only published case [9]. One of the largest literature reviews on the topic of primary mediastinal seminomas, was published in 1984 by Aygun et al. which presented 3 new cases and reviewed 124 existing cases in the literature; a total of which 74 where managed by surgery [11]. The number of reported cases of primary mediastinal seminoma found incidentally following surgery are small. 1–4% of all mediastinal masses are germ cell in origin with the majority of cases being teratomas and not seminomas [1, 2].

Also histologically, the background of florid follicular lymphoid hyperplasia, as seen in this particular case, is very rare. A paper by Weissferdt et al [13] looked at 6 cases showing a similar pattern. In this paper, it is suggested that the background of florid lymphoid tissue may represent a prominent immune response. In all 6 cases the same immumohistolchemical stains were used to diagnose a seminoma, such as OCT 4 and PLAP. Follicular hyperplasia was seen in all cases with the same staining, such as CD20 and CD3. As shown in Table 1 histologically mediastinal seminomas can be associated with secondary features such as remnant thymic tissue, non-caseating epithelioid granulomas cysts, and fibrosis. These tumours as in our case were associated with reactive follicular lymphoid hyperplasia. The secondary features and reactive hyperplasia, when pronounced, can obscure the malignant seminomatous component and the tumour can be mistaken for a reactive condition or benign lesion [13–15].

**Table 1:** Clinical and histological features of 6 cases described by Weissferdt et al [13] all patient had been diagnosed with an anterior mediastinal mass found to be a primary mediastinal seminoma with follicular hyperplasia on histological examination with the same immunohistology stains as in our case

Case	Gender	Age	Tumour size (cm)	Cystic changes	Epithelioid granulomas	Thymic remnant/Hassall’s corpuscles	Foreign body type giant cells	Calcifications	Cholesterol clefts	Necrosis
1	Male	28	5	–	–	+	+	+	+	+
2	Male	24	3	+	+	+	+	–	–	–
3	Male	29	5	+	+	+	+	–	+	–
4	Male	25	4	–	–	+	+	–	–	–
5	Male	31	3.5	+	+	–	+	+	–	–
6	Male	24	3.5	+	+	–	+	–	–	–

At clinical follow up, 5 of the patients in the paper were alive and well 3 to 5 years after diagnosis; one patient was lost to follow up. The conclusion of this paper was that the true prognosis of these pattern of mediastinal seminoma with florid lymphoid hyperplasia needs to be investigated in a larger case series [13].

At the time of writing of this paper, there were only three further publications that had reported similar cases. In the 2016, paper by Lee et al. had described a single case with the same immuohistological features, except for the presence of a synchronous thymoma [14]. And finally back in 1986, a paper by Burns et al. described three cases all with the same histological features as our case and the case series by Weissferdt et al. [13, 15] Therefore, to date, including our current case, a total of 11 cases of mediastinal seminoma with florid lymphoid hyperplasia have been reported [13–15].

First line treatment in resectable mediastinal seminomas is surgical excision, with possible radiotherapy post-operatively depending on the status of histological margins [2, 3, 6, 11]. Surgical approaches could include thoracoscopic or robotic and thoracotomy or sternotomy. In our case, due to the size of the tumour and mediastinal fat, and patient’s body habitus, it was decided robotic and thoracoscopic approaches would have a high risk of conversion to open. Good exposure is the main goal, to obtain a good margin whilst conserving the innominate vein, and phrenic nerves [3, 6, 7, 10, 11]. Pure seminomas are radiotherapy and chemotherapy sensitive and respond well to this treatment, if deemed not suitable for surgery. In summary, the overall prognosis is similar to gonadal seminomas with a 5 year survival of 87–100% [1–4, 7].

## Data Availability

Not applicable.

## Abbreviations

αFP: Alpha-fetoprotein
βHCG: Beta human chorionic gonadotropin
BMI: Body mass index
CT: Computed tomography
LDH: Lactate dehydrogenase
MDT: Multidisciplinary team
MRI: Magnetic resonance imaging
PET: Positive emission tomography
PLAP: Placental alkanie phosphatase
TdT: Terminal deoxynucleotidyl transferase
V/Q: Ventilation perfusion

## References

[1] Gupta D, Rath A, Rathi KR, Singh G (2016). Primary thymic mediastinal seminoma with florid granulomatous reaction. Indian J Pathol Microbiol.

[2] Napieralska A, Majewski W, Osewski W, Miszczyk L (2018). Primary mediastinal seminoma. J Thor Dis.

[3] Petrova D, Kraleva S, Muratovska L, Crcareva B (2019). Primary seminoma localized in mediastinum: case report. Open Access Macedonian J Med Sci.

[4] Woolner LB, Jamplis RW, Kirklin JW (1955). Seminoma (germinoma) apparently primary in the anterior mediastinum. N Engl J Med.

[5] WHO Classification Editorial Board (2021). Thoracic tumours.

[6] Bishop MA, Kyriakopoulos C (2020). Mediastinal seminoma. StatPearls.

[7] Kermenli T, Azar C (2019). Evaluation of surgical procedures in primary mediastinal cysts and tumors: single-center experience. Kardiochirurgia i Torakochirurgia Polska.

[8] Imai T, Morishita Y, Ito S (2020). A case of ectopic thyroid presenting as a superior Mediastinal mass. Cureus.

[9] Chen ZG, Pan HX, Wang T, Cai LE, Lei YY, Su CH (2010). Primary thymic seminoma in a 32-year-old female. Ann Oncol.

[10] Li WW, van Boven WJ, Annema JT, Eberl S, Klomp HM, de Mol BA (2016). Management of large mediastinal masses: surgical and anesthesiological considerations. J Thoracic Dis.

[11] Aygun C, Slawson RG, Bajaj K, Salazar O (1984). Primary mediastinal seminoma. Urology..

[12] Thompson MK, Lynskey DM, Doherty GJ. Primary Mediastinal seminoma presenting with superior vena cava obstruction. BMJ Case Rep. 2017;2017. Published online first 25 July 2017. Available from https://casereports.bmj.com/content/2017/bcr-2017-221071. 10.1136/bcr-2017-221071 PMC5589070 28855218

[13] Weissferdt A, Moran CA (2015). Mediastinal seminoma with florid follicular lymphoid hyperplasia: a clinicopathological and immunohistochemical study of six cases. Virchows Arch.

[14] Lee HI, Jang IS, Jeon KN, Ko GH, Lee JS, Kim DC (2017). Thymoma and synchronous primary Mediastinal seminomas with florid follicular lymphoid hyperplasia in the anterior mediastinum: a case report and review of the literature. J Pathol Transl Med.

[15] Burns BF, McCaughey WT (1986). Unusual thymic seminomas. Arch Pathol Lab Med.

